# Supplementary material for: Arctiin elevates osteogenic differentiation of MC3T3-E1 cells by modulating cyclin D1
Source: Bioengineered. 2022 Apr 27;13(4):10866–74. doi: 10.1080/21655979.2022.2066047 (PMC9208514; doi:10.1080/21655979.2022.2066047)
Supplement: Supplemental Material [file KBIE_A_2066047_SM4351.zip › supplementary/Revised_manuscript_untracked.docx]

**Arctiin elevates osteogenic differentiation of** **MC3T3-E1 cells** **by modulating cyclin D1**

Ziye Liu^1^, Yongsheng Wu^2, #^

^1^Department of Joint Surgery and Sports Medicine, Shanghai Changzheng Hospital, Naval Medical University, Shanghai, 200001, China.

^2^Second Department of Orthopaedics, Zhuhai Hospital of Guangdong Provincial Hospital of traditional Chinese Medicine, Zhuhai City, Guangdong Province, 519000, China.

^#^Corresponding author: Yongsheng Wu, Address: Second Department of Orthopaedics, Zhuhai Hospital of Guangdong Provincial Hospital of traditional Chinese Medicine, No.53, Jida jingle Road, Xiangzhou District, Zhuhai City, Guangdong Province, 519000, China.

E-mail: [wyswyssunlight@163.com](mailto:wyswyssunlight@163.com)

Running title: Effects of Arctiin on osteogenic differentiation

**Abstract**

Osteoporosis is a systemic disorder of bone metabolism. This study aimed to investigate the impacts and possible mechanisms of Arctiin, a lignin isolated from *Arctium lappa* on MC3T3-E1 osteoblast differentiation. In this study, after treatment with different concentrations of Arctiin, reverse transcription-quantitative polymerase chain reaction (RT-qPCR) and western blotting were to estimate the expression of osteogenesis markers. Then, the activity of alkaline phosphatase (ALP) was detected by an ALP assay kit and calcium nodules staining was evaluated by alizarin red staining (ARS). Additionally, the regulatory effects of Arctiin on cyclin D1 (Ccnd1) was assessed by measurement of protein expression. Subsequently, the functions of Ccnd1 silencing on the osteogenic differentiation was examined in Arctiin-treated MC3T3-E1 cells. Results indicated that Arctiin dose-dependently upregulated the expression of runt-related transcription factor 2 (RUNX2), collagen type 1 (COL1A1), osteocalcin (OCN) and osteopontin (OPN). Elevated ALP activity and calcification degree was prominently observed in the Arctiin-treated groups. Moreover, Ccnd1 expression was notably enhanced after Arctiin intervention. Importantly, Ccnd1-knockdown abrogated the impacts of Arctiin on osteogenic differentiation of MC3T3-E1. To conclude, findings in this study suggested that Arctiin could regulate MC3T3-E1 osteoblast differentiation via up-regulating Ccnd1, supporting that Arctiin might be a therapeutic target for osteoporosis.

**Keywords:** osteogenic differentiation, Arctiin, Ccnd1, alkaline phosphatase

**Introduction**

Osteoporosis is a frequent disease in bone characterized by bone weakening, low bone mineral density and bone mass, which contributes to a decrease in bone strength and ultimately leads to increased risk of fracture (1). The high morbidity of osteoporotic fractures and the economic burden it impose have increasingly brought to public attention (2). In recent years, osteoporosis has gradually become an urgent public health issue affecting millions of aging people worldwide (3). Osteoporotic fracture is a serious consequence of osteoporosis, vertebral body is the most common fracture site. Patients with spinal osteoporosis fracture suffer from the end of the patient's functional independence and labor incapacity, which results in the reduced quality of life and increased heavy economic burden (4). Thus, the identification of novel agents associated with osteoporosis is of clinical significance.

Osteogenic differentiation is deemed as a pivotal determinant in bone regeneration. Accumulating literature has supported that abnormal bone remodeling and osteoblastic bone formation are responsible for the initiation and development of osteoporosis (5). It is considered as an indispensable therapeutic strategy for osteoporosis to increase bone formation. Arctiin, commonly called greater burdock, is a lignin bioactive compound extracted from the dried ripe fruit of *Arctium lappa L* (6). A considerable body of evidence indicates that Arctiin plays a crucial role in exerting anti-viral, anti-tumor and anti-inflammation properties (6-8). Emerging evidence supports the notion that Arctiin blocks osteoclastogenesis and bone resorption via the suppression of receptor activator of nuclear factor-κB ligand (RANKL)-induced reactive oxygen species (9). Osteoporosis is caused by deficiency in bone formation by osteoblasts and excessive bone resorption by osteoclasts (10, 11). Therefore, whether Arctiin can regulate osteogenic differentiation and the potential regulatory mechanisms remain to be elucidated.

This paper was intended to clarify the roles of Arctiin in the capacity of mouse pre-osteoblast MC3T3-E1 to differentiate into osteoblast, and further explored the regulatory effects of Arctiin on cyclin D1 (Ccnd1) expression. These results might provide novel insight into anti-osteoporosis effect of Arctiin.

**Materials and methods**

**Culture and treatment of MC3T3-E1 cells**

Murine preosteoblast MC3T3-E1 cells acquired from Chinese Academy of Sciences (Shanghai, China) were maintained in Minimum Essential Medium α (α-MEM; HYClone) medium followed by the addition of 10% fetal bovine serum (FBS; Biological Industries) under the condition of 37 °C and 5% CO_2_. For osteoblastic differentiation experiments, to induce osteogenic differentiation, cells were cultivated in α-MEM supplemented with 10% FBS, 20 mM beta-glycerophosphate and 100 mg/mL ascorbic acid (Sigma-Aldsrich; Merck KGaA) for a longest period of 14 days (12). Different concentrations of Arctiin (2.5, 5, 10 and 20 μM) were employed to treat MC3T3-E1 cells for 48 h (9). Arctiin of purity > 98% was obtained from Yuanye biological company (Shanghai, China).

**STITCH analysis**

The STITCH website (<http://stitch.embl.de/>) was used to predict the possible interactions among Arctiin and proteins using Mus musculus as an organism. The protein had higher score and related to the progression of osteoporosis was selected to perform the further experiments.

**Cell Counting Kit-8 (CCK-8) assay**

The cell viability assay was conducted using a cell counting kit-8 kit (CCK-8; Shanghai Yi Sheng Biotechnology Co. Ltd., China). Various concentrations of Arctiin (2.5, 5, 10 and 20 μM) were utilized to treat MC3T3-E1 (2×10^3^ cells/well) osteoblasts plated into 96-well plates at 37 °C for 48 h. Then, a microplate reader (Molecular Devices, Sunnyvale, CA) was applied to record the absorbance at 450 nm 4 h later after CCK-8 solution (10 μL) was jointed to each well.

**Alkaline phosphatase (ALP) activity assay**

ALP activity was appraised by an ALP assay kit (Sigma, St. Louis, USA) according to the standard protocol. Brieﬂy, MC3T3-E1 cells (1×10^5^ cells/well) inoculated in 24-well plates were exposed to osteogenic induction medium for two weeks. The obtained lysates by adioimmunoprecipitation (RIPA) buffer (Sigma-Aldrich) were centrifugated at 200 x g for 5 min. A microplate reader (Molecular Devices, Sunnyvale, CA) was to calculate ALP activity using the optical density (OD) at 405.

**Alizarin red staining (ARS)**

ARS assay was to examine mineralization nodule formation (13). MC3T3-E1 cells were seeded in 6-well plates (2×10^5^ cells per well) and osteogenesis was determined at 14 days in osteogenic medium with or without Arctiin. After fixation in 4% paraformaldehyde for 30 min, cells were washed by PBS. Then, cells were stained by 1% ARS solution (Sigma-Aldrich) for 15 min and a light microscope (OLYMPUS, Japan) was employed to photograph stained cells. To quantify mineralization, calcium deposits were desorbed using 10% cetylpyridinium chloride (Sigma-Aldrich; Merck KGaA) for 30 min, and the absorbance at 562 nm was measured in in a plate reader (Molecular Devices, Sunnyvale, CA).

**Cell transfection**

Short hairpin RNA (shRNA) targeting Ccnd1 (shCcnd1; 5’-GCCACAGATGTGAAGTTCA-3’) and its negative control (shNC) were purchased from GenePharma (Shanghai, China). MC3T3-E1 cells grown on 6-well plates were transfected with shCcnd1 or shNC by means of lipofectamine 2000 reagent (Invitrogen, Carlsbad, USA) in accordance with the manufacturer's guidelines. At 48 h after post-transfection, the transfection efficiency was tested.

**Western blot analysis**

The concentration of isolated proteins by RIPA buffer (Sigma-Aldrich) was quantified by a bicinchoninic acid protein assay kit (BestBio, Inc.). Following, 40 μg of proteins per lane were applied to 10% sodium dodecyl sulfate-polyacrylamide gel electrophoresis (SDS-PAGE) and loaded onto polyvinylidene fluoride membrane (PVDF; Bio-Rad, Hercules, CA, USA). A total of 5% fat-free milk solution was used to block these blots. The blots were incubated with specific primary antibodies overnight at 4°C, followed by incubation of horseradish peroxide (HRP)-conjugated secondary antibody at room temperature for 1.5 h. Immune complexes were recognized with an enhanced chemiluminescence reagent (EMD Millipore). Image Lab™ Software (Bio‐Rad, Hercules, CA) was employed to evaluate the band density. The relative protein expression was calculated based on glyceraldehyde-phosphate dehydrogenase (GAPDH) as the loading control. The primary antibodies used in this study included anti-runt-related transcription factor 2 (RUNX2; 12556S, 1:1000, Cell Signaling Technology, Boston, MA, USA), anti-collagen type 1 (COL1A1; 72026T, 1:1000, Cell Signaling Technology, Boston, MA, USA), anti-osteocalcin (OCN; ab93876, 1:1000, Abcam, Cambridge, UK), anti-osteopontin (OPN; ab283656, 1:1000, Abcam, Cambridge, UK), anti-Ccnd1 (55506T, 1:1000, Cell Signaling Technology, Boston, MA, USA) and anti-GAPDH (5174T, 1:1000, Cell Signaling Technology, Boston, MA, USA).

**Reverse transcription-quantitative polymerase chain reaction (RT-qPCR) analysis**

The isolation of total RNA was conducted by TRIzol^®^ reagent (Invitrogen, NY, USA). Then, the complementary DNA (cDNA) was prepared using RT reactions with a first Strand cDNA Synthesis Kit (TakaRa, Dalian, China). An ABI 7500 Thermocycler (Applied Biosystems; Thermo Fisher Scientific, Inc.) was introduced to qPCR implementation. The primers used in this study were provided by Sangon Biotech. Fold changes of gene expression was determined based on the 2^-ΔΔCq^ method (14)(15). GAPDH was served as an internal control. The primer sequences used in this study are shown in Table 1.

**Statistical analysis**

All experiments were repeated independently in triplicate. Values are presented as mean ± standard deviation and analysis was performed using GraphPad Prism 6.0 (GraphPad Software, Inc.). Differences among groups were estimated with the aid of one-way analysis of variance (ANOVA) followed by Tukey's post hoc test. A value of p < 0.05 was considered as a statistically significant result.

**Results**

**Arctiin increased the expression levels of bone formation markers in MC3T3-E1 cells**

As a lignin bioactive compound, Arctiin has been reported to block osteoclastogenesis and bone resorption via the suppression of RANKL-induced reactive oxygen species (9). However, whether Arctiin could regulate osteogenic differentiation in MC3T3-E1 cells remain to be elucidated. Firstly, CCK-8 assay was to appraise the viability of MC3T3-E1 cells after different concentrations of Arctiin treatment. As displayed in **Fig. 1A**, Arctiin concentrations of 2.5, 5 and 10 μM had no significant difference in the viability of MC3T3-E1 cells compared with the untreated control group. However, a notably decrease on cell proliferation was noticed when 20 μM of Arctiin was utilized to treat MC3T3-E1 cells. Therefore, 2.5, 5 and 10 μM Arctiin were used to perform the following experiments. As what is observable from **Fig. 1B-C**, Arctiin dose-dependently unregulated the expression of osteogenesis markers including RUNX2, COL1A1, OCN and OPN in the transcriptional and post-transcriptional levels as comparison to the control group. These results suggest that Arctiin accelerates the expression of bone formation markers in MC3T3-E1 cells.

**Arctiin promoted the** **osteogenic differentiation in MC3T3-E1 cells**

Osteogenic differentiation is deemed as a pivotal determinant in bone regeneration, which is considered as an indispensable therapeutic strategy for osteoporosis (15, 16). Afterwards, the activity of ALP, a ubiquitous cellular protein and bone formation marker that is closely related to the early osteogenic differentiation, was evaluated with a commercial available kit. As exhibited in **Fig. 2A**, Arctiin elevated the activity of ALP in a concentration-dependently manner. Additionally, result of alizarin red staining displayed in **Fig. 2B-C** indicated that Arctiin intervention remarkably enhanced the ability of mineralization in MC3T3-E1 cells relative to the untreated control group, and the highest level of calcium deposit was found in the 10 μM Arctiin-treated group. These observations reveal that Arctiin promotes the differentiative capacity of MC3T3-E1 cells into osteoblasts.

**Arctiin could upregulate Ccnd1 expression in MC3T3-E1 cells**

To explore the potential regulatory mechanisms of Arctiin in the osteogenic differentiation of MC3T3-E1 cells, the STITCH website (<http://stitch.embl.de/>) was used to detect the possible targets of Arctiin. It was found that Arctiin could bind to Ccnd1 and had higher score (**Fig. 3A**). Besides, result of **Fig. 3B** suggested that Arctiin dose-dependently upregulated the expression of Ccnd1 relative to the control group. Through the above findings we proved that Arctiin can upregulate Ccnd1 expression in MC3T3-E1 cells.

**Ccnd1 silencing restored the effects of Arctiin on MC3T3-E1 cell osteogenicity**

Subsequently, Ccnd1 was silenced by transfection with shRNA targeting Ccnd1 to further explore whether Arctiin elevates osteogenic differentiation of MC3T3-E1 cells by modulating cyclin D1 expression. Significantly reduced expression of Ccnd1 was observed in the Arctiin plus shCcnd1 group as comparison to the Arctiin plus shNC group (**Fig. 4A**). Moreover, the expression of osteogenesis markers was detected. As displayed in **Fig. 4B-D**, RUNX2, COL1A1, OCN and OPN expression at mRNA level and protein level were conspicuously decreased after Ccnd1 was knocked down in MC3T3-E1 cells stimulated by Arctiin relative to the shNC group. Concurrently, lose-function of Ccnd1 remarkably reduced the activity of ALP and the degree of mineralization in MC3T3-E1 cells with Arctiin intervention (**Fig. 5A-C**). To be summarized, these findings provide evidence that Arctiin promotes the differentiation of MC3T3-E1 cells into osteogenic lineages through mediating Ccnd1.

**Discussion**

Research has proposed that bone remodeling plays a crucial role in the maintenance and regeneration of bone tissues (17, 18). The disturbance of bone remodeling results in bone abnormalities, of which the most common one is osteoporosis. It’s widely accepted that osteoblast is the sole bone-forming cell, which plays a critical role in bone remodeling and osteoblastic bone formation. Chinese herbal medicine display a promising therapy for maintaining bone health and preventing bone disorders (19, 20). In the present study, the effects of Arctiin, a lignin bioactive compound extracted from the dried ripe fruit of *Arctium lappa L*, on osteoblastic MC3T3-E1 cells *in vitro* were assessed. It was discovered that Arctiin could exacerbate the ability of MC3T3-E1 cells to differentiate towards osteogenic lineages by targeting Ccnd1.

Osteoblast differentiation and mineralization are important stages in bone formation (21, 22). Emerging factors participates in this process of bone formation and regeneration. RUNX2 is a master osteoblast lineage-determining transcription factor, which is closely associated to control osteoblastic differentiation, matrix production, and mineralization (23, 24). RUNX2 is considered to be the master gene in osteogenesis for its capacity to trigger the expression of osteoblast-specific genes, including OCN and OPN (25). Besides, COL1A1 is a significant extracellular matrix protein, which provides structural support, cell migration anchorage and cell differentiation cues during bone regeneration (26). Additionally, ALP can hydrolyze various types of phosphates to promote cell maturation and calcification (27). It is worthy of note that Arctiin blocks osteoclastogenesis and bone resorption via suppressing RANKL-induced reactive oxygen species (9). Our study initially demonstrated that Arctiin could upregulate the expression levels of aforementioned osteoblast-related genes.

Figuring out the mechanisms underlying osteoblast differentiation and calcification is critical to improve the therapy for osteoporosis. To elucidate the potential mechanism of Arctiin functioning in the regulation of MC3T3-E1 cell differentiation, the STITCH website (<http://stitch.embl.de/>) was used to predict the possible targets of Arctiin. It was found that Arctiin could bind to Ccnd1. Ccnd1, the abbreviation of cyclin D1 (Mus musculus), is located on chromosome 7 and encoded by mouse Ccnd1 gene. A previous study reported that Ccnd1 was targeted by Let-7b to modulate osteoblast differentiation in mouse MC3T3-E1 cells (28). MiR-23b-3p functions as a positive factor for the progression of osteoporosis via targeting Ccnd1 in MC3T3-E1 cells (29). Ccnd1 expression was elevated by miR-539 up-regulation in osteoblasts (30). Moreover, an existing study has shown that miR-34a/Ccnd1 axis contributed to osteogenic differentiation (31). In this study, Ccnd1 was demonstrated to be regulated by Arctiin. And Ccnd1 silencing partially counteracted the promoting effects of Arctiin on MC3T3-E1 cells’ osteogenic potential.

Taken together, this is the first study to show that the impacts of Arctiin on MC3T3-E1 osteoblast differentiation. Mechanically, we validated that Arctiin promoted MC3T3-E1 cells’ osteogenic differentiation ability by targeting Ccnd1. Our findings provides experimental supports for the application of Arctiin in improving new bone formation and treating pathological conditions of bone loss. As Ccnd1 is a target of various pathways, the next experiments will investigate the effects on these pathways by Arctiin. Additionally, we will make the animal experiment in the future study to enhance the reliability of experimental results and provide theoretical basis for clinical application.

**Acknowledgements**

Not applicable.

**Declaration of interest statement**

The authors declare that they have no competing interests.

**Funding**

Not applicable.

**Availability of data and materials**

The experimental data will be available from the corresponding author on reasonable request.

**References**

1. Chai XY, Zhang WC, Chang BY, et al.: GPR39 agonist TC-G 1008 promotes osteoblast differentiation and mineralization in MC3T3-E1 cells. Artif. Cell. Nanomed. Biotechnol. 47: 3569-3576, 2019.

2. Tella SH and Gallagher JC: Prevention and treatment of postmenopausal osteoporosis. J Steroid Biochem Mol Biol 142: 155-170, 2014.

3. Pavone V, Testa G, Giardina SMC, Vescio A, Restivo DA and Sessa G: Pharmacological Therapy of Osteoporosis: A Systematic Current Review of Literature. Front Pharmacol 8: 803, 2017.

4. Wang Q, Wang S, Li XZ, Wei YC and Ma XQ: Clinical observation of percutaneous vertebroplasty in the treatment of senile osteoporosis complicated with vertebral compression fractures. Int. J. Clin. Exp. Med. 11: 7340-7345, 2018.

5. Weitzmann MN and Ofotokun I: Physiological and pathophysiological bone turnover - role of the immune system. Nat Rev Endocrinol 12: 518-532, 2016.

6. Lee JH, Kim C, Lee J, Um JY, Sethi G and Ahn KS: Arctiin is a pharmacological inhibitor of STAT3 phosphorylation at tyrosine 705 residue and potentiates bortezomib-induced apoptotic and anti-angiogenic effects in human multiple myeloma cells. Phytomedicine 55: 282-292, 2019.

7. Hayashi K, Narutaki K, Nagaoka Y, Hayashi T and Uesato S: Therapeutic effect of arctiin and arctigenin in immunocompetent and immunocompromised mice infected with influenza A virus. Biol Pharm Bull 33: 1199-1205, 2010.

8. Zhou B, Weng G, Huang Z, Liu T and Dai F: Arctiin Prevents LPS-Induced Acute Lung Injury via Inhibition of PI3K/AKT Signaling Pathway in Mice. Inflammation 41: 2129-2135, 2018.

9. Chen D, Ye Z, Wang C, et al.: Arctiin abrogates osteoclastogenesis and bone resorption via suppressing RANKL-induced ROS and NFATc1 activation. Pharmacol Res 159: 104944, 2020.

10. Ren Y, Zhang K, Wang J, et al.: HOTAIRM1 promotes osteogenic differentiation and alleviates osteoclast differentiation by inactivating the NF-kappaB pathway. Acta Biochim Biophys Sin (Shanghai) 53: 201-211, 2021.

11. Sozen T, Ozisik L and Basaran NC: An overview and management of osteoporosis. Eur J Rheumatol 4: 46-56, 2017.

12. Jiang F, Shan H, Pan C, et al.: ATP6V1H facilitates osteogenic differentiation in MC3T3-E1 cells via Akt/GSK3beta signaling pathway. Organogenesis 15: 43-54, 2019.

13. Chen X, Liu Y, Meng B, Wu D, Wu Y and Cao Y: Interleukin-20 inhibits the osteogenic differentiation of MC3T3-E1 cells via the GSK3beta/beta-catenin signalling pathway. Arch Oral Biol 125: 105111, 2021.

14. Livak KJ and Schmittgen TD: Analysis of relative gene expression data using real-time quantitative PCR and the 2(-Delta Delta C(T)) Method. Methods (San Diego, Calif.) 25: 402-408, 2001.

15. Li W, Zhang S, Liu J, Liu Y and Liang Q: Vitamin K2 stimulates MC3T3E1 osteoblast differentiation and mineralization through autophagy induction. Mol Med Rep 19: 3676-3684, 2019.

16. Ahn GY, Kim SE, Yun TH, Choi I, Park D and Choi SW: Enhanced osteogenic differentiation of alendronate-conjugated nanodiamonds for potential osteoporosis treatment. Biomater Res 25: 28, 2021.

17. Hasegawa T and Amizuka N: [Bone remodeling and modeling/mini-modeling.]. Clin Calcium 27: 1713-1722, 2017.

18. Jimi E: [The role of osteoclastic bone resorption on bone remodeling.]. Clin Calcium 27: 1689-1695, 2017.

19. Putnam SE, Scutt AM, Bicknell K, Priestley CM and Williamson EM: Natural products as alternative treatments for metabolic bone disorders and for maintenance of bone health. Phytother. Res. 21: 99-112, 2007.

20. An J, Yang H, Zhang Q, et al.: Natural products for treatment of osteoporosis: The effects and mechanisms on promoting osteoblast-mediated bone formation. Life Sci. 147: 46-58, 2016.

21. Wang Q, Xie X, Zhang D, Mao F, Wang S and Liao Y: Saxagliptin enhances osteogenic differentiation in MC3T3-E1 cells, dependent on the activation of AMP-activated protein kinase alpha (AMPKalpha)/runt-related transcription factor-2 (Runx-2). Bioengineered 13: 431-439, 2022.

22. Ji W and Sun X: Methyl-CpG-binding protein 2 promotes osteogenic differentiation of bone marrow mesenchymal stem cells through regulating forkhead box F1/Wnt/beta-Catenin axis. Bioengineered 13: 583-592, 2022.

23. Sun M, Chi G, Li P, et al.: Effects of Matrix Stiffness on the Morphology, Adhesion, Proliferation and Osteogenic Differentiation of Mesenchymal Stem Cells. Int J Med Sci 15: 257-268, 2018.

24. Liu TM and Lee EH: Transcriptional regulatory cascades in Runx2-dependent bone development. Tissue Eng Part B Rev 19: 254-263, 2013.

25. Jeong JH and Choi JY: Interrelationship of Runx2 and estrogen pathway in skeletal tissues. BMB Rep 44: 613-618, 2011.

26. Shang F, Ming L, Zhou Z, et al.: The effect of licochalcone A on cell-aggregates ECM secretion and osteogenic differentiation during bone formation in metaphyseal defects in ovariectomized rats. Biomaterials 35: 2789-2797, 2014.

27. Zhang JF, Zhang WB, Dai JW, Wang XD and Shen SGF: Overexpression of Dlx2 enhances osteogenic differentiation of BMSCs and MC3T3-E1 cells via direct upregulation of Osteocalcin and Alp. Int. J. Oral Sci. 11: 12, 2019.

28. Wang LJ and Cai HQ: Let-7b downgradesCCND1to repress osteogenic proliferation and differentiation ofMC3T3-E1cells: An implication in osteoporosis. Kaohsiung J. Med. Sci. 36: 775-785, 2020.

29. Wang J and Zhao B: MiR-23b-3p functions as a positive factor for osteoporosis progression by targeting CCND1 in MC3T3-E1 cells. In vitro cellular & developmental biology. Animal 57: 324-331, 2021.

30. Zhu X, Lin W, Lv C, et al.: MicroRNA-539 promotes osteoblast proliferation and differentiation and osteoclast apoptosis through the AXNA-dependent Wnt signaling pathway in osteoporotic rats. Journal of cellular biochemistry 119: 8346-8358, 2018.

31. Fan C, Jia L, Zheng Y, et al.: MiR-34a Promotes Osteogenic Differentiation of Human Adipose-Derived Stem Cells via the RBP2/NOTCH1/CYCLIN D1 Coregulatory Network. Stem Cell Reports 7: 236-248, 2016.

**Figure legends**

Figure 1. Arctiin elevated bone formation in MC3T3-E1 cells. (A) Cell viability was determined with a CCK-8 assay after the transfected or non-transfected MC3T3-E1 cells (2×10^3^ cells/well) treatment with various concentrations of Arctiin (2.5, 5, 10 and 20 μM) for 48 h. (B) RT-qPCR and (C) western blot were employed to examine the expression of osteogenesis markers including RUNX2, COL1A1, OCN and OPN. ^**^P < 0.01, ^***^P < 0.001.

Figure 2. Arctiin promoted the osteogenic differentiation in MC3T3-E1 cells. (A) The viability of ALP was evaluated using an ALP assay kit. (B-C) ARS was employed to detect the level of calcification. ^**^P < 0.01, ^***^P < 0.001.

Figure 3. Arctiin could upregulate Ccnd1 expression in MC3T3-E1 cells. (A) The STITCH website (<http://stitch.embl.de/>) was used to predict the bind of Arctiin to Ccnd1. (B) The expression of Ccnd1 was measured by means of western blot analysis. ^***^P < 0.001.

Figure 4. Ccnd1 downregulation attenuated the impacts of Arctiin on bone formation in MC3T3-E1 cells. (A) The expression of Ccnd1 was determined with western blotting after transfection with shRNA targeting Ccnd1. (B) The mRNA expression of RUNX2, COL1A1, OCN and OPN was detected using RT-qPCR. (C-D) Western blot analysis was adopted for the assessment of RUNX2, COL1A1, OCN and OPN proteins expression. ^*^P < 0.05, ^**^P < 0.01, ^***^P < 0.001.

Figure 5. Ccnd1 silencing blocked the impacts of Arctiin on osteogenic differentiation in MC3T3-E1 cells. (A) ALP activity was evaluated using an ALP assay kit. (B-C) ARS measured the level of calcification. ^***^P < 0.001.
