# Supplementary figures and images for: Arctiin elevates osteogenic differentiation of MC3T3-E1 cells by modulating cyclin D1
Source: Bioengineered. 2022 Apr 27;13(4):10866–74. doi: 10.1080/21655979.2022.2066047 (PMC9208514; doi:10.1080/21655979.2022.2066047)

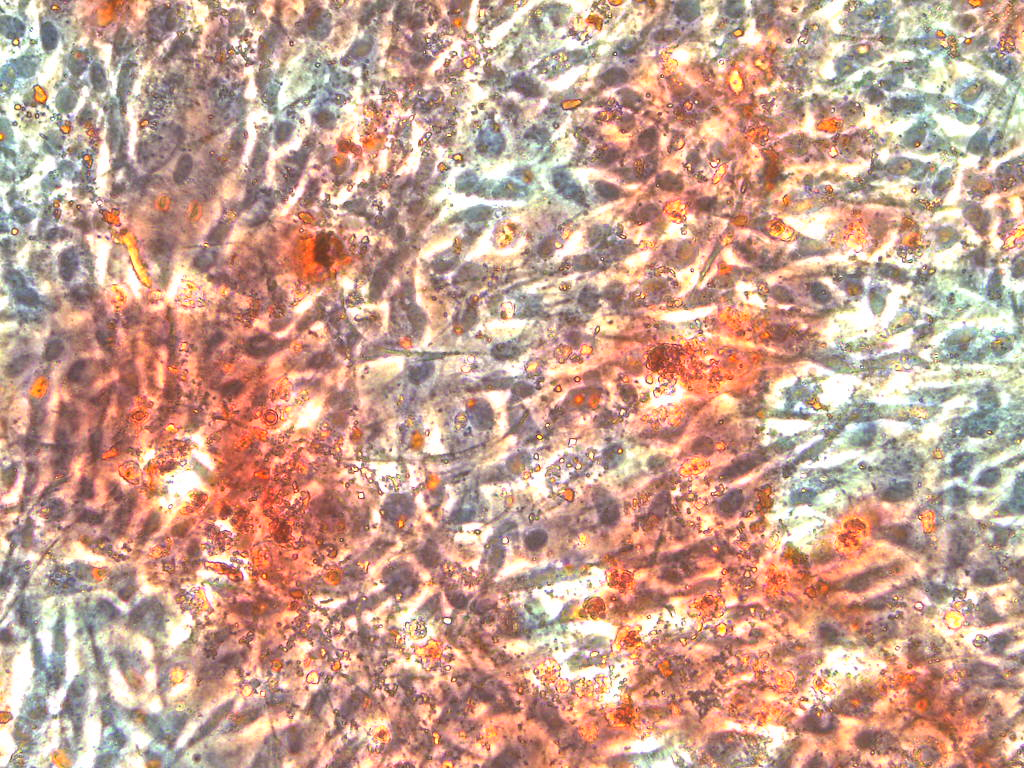

Supplement: Supplemental Material [file KBIE_A_2066047_SM4351.zip › supplementary/Fig2B_10uM.jpg]

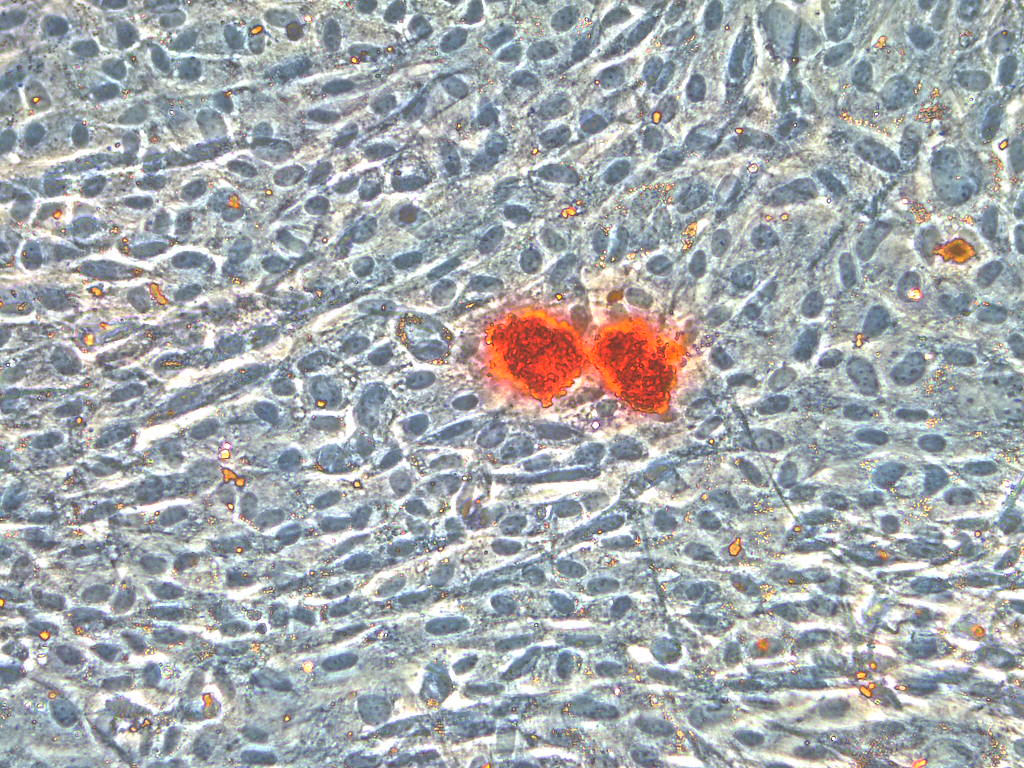

Supplement: Supplemental Material [file KBIE_A_2066047_SM4351.zip › supplementary/Fig2B_2_5uM.jpg]

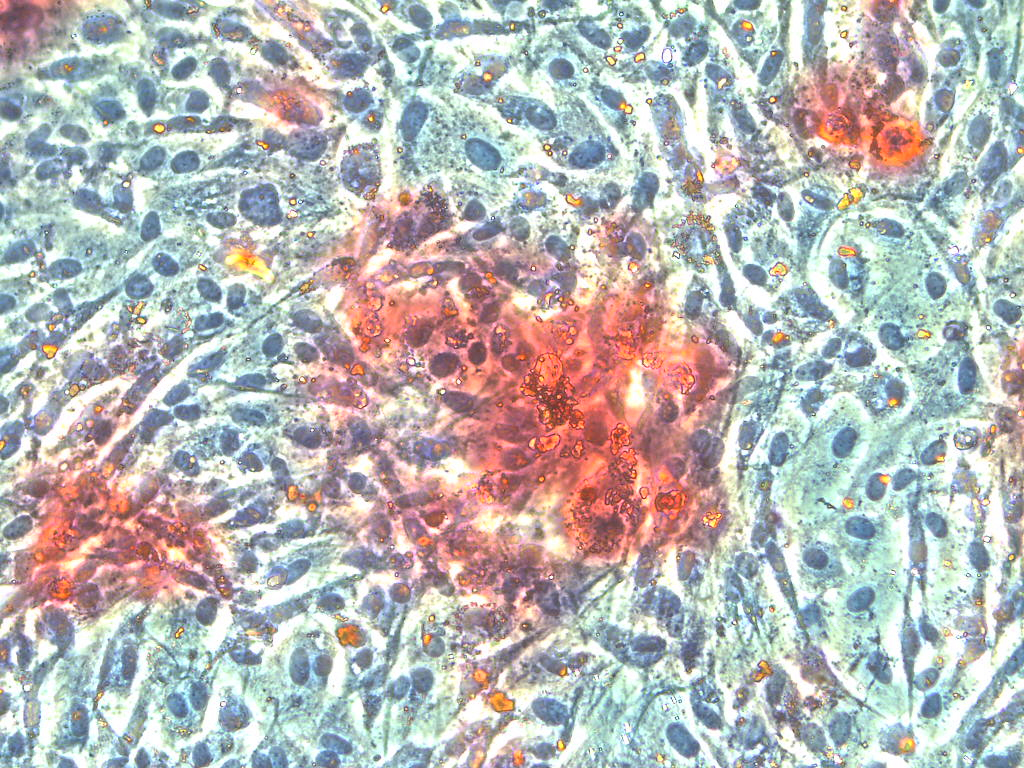

Supplement: Supplemental Material [file KBIE_A_2066047_SM4351.zip › supplementary/Fig2B_5uM.jpg]

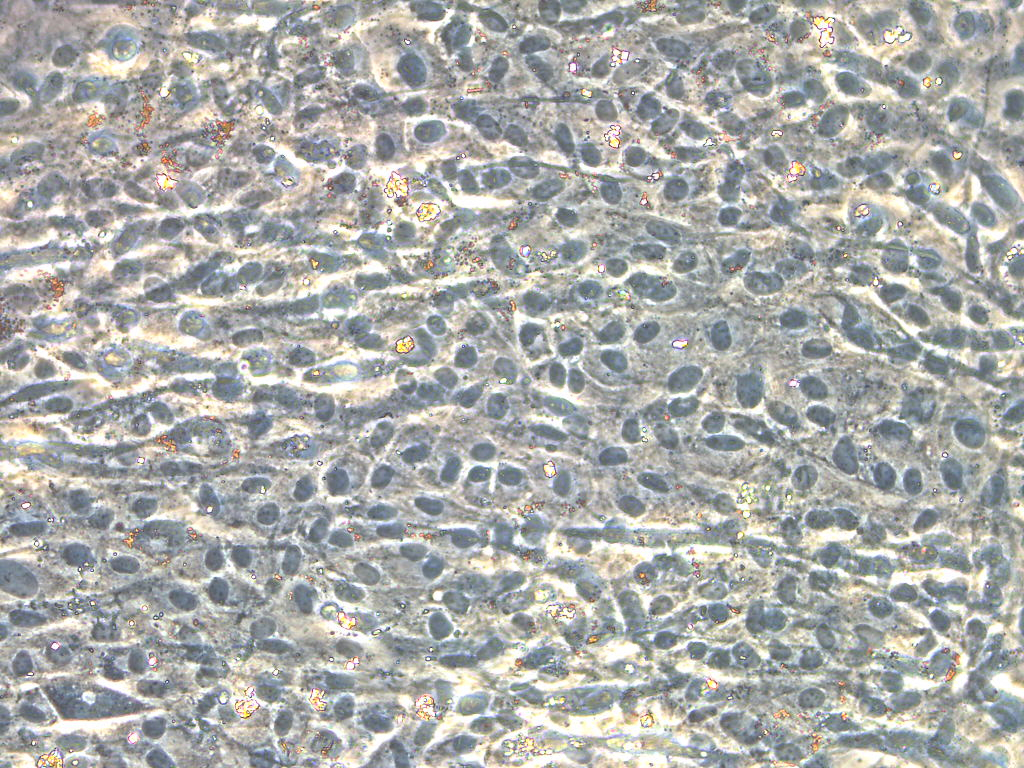

Supplement: Supplemental Material [file KBIE_A_2066047_SM4351.zip › supplementary/Fig2B_Control.jpg]

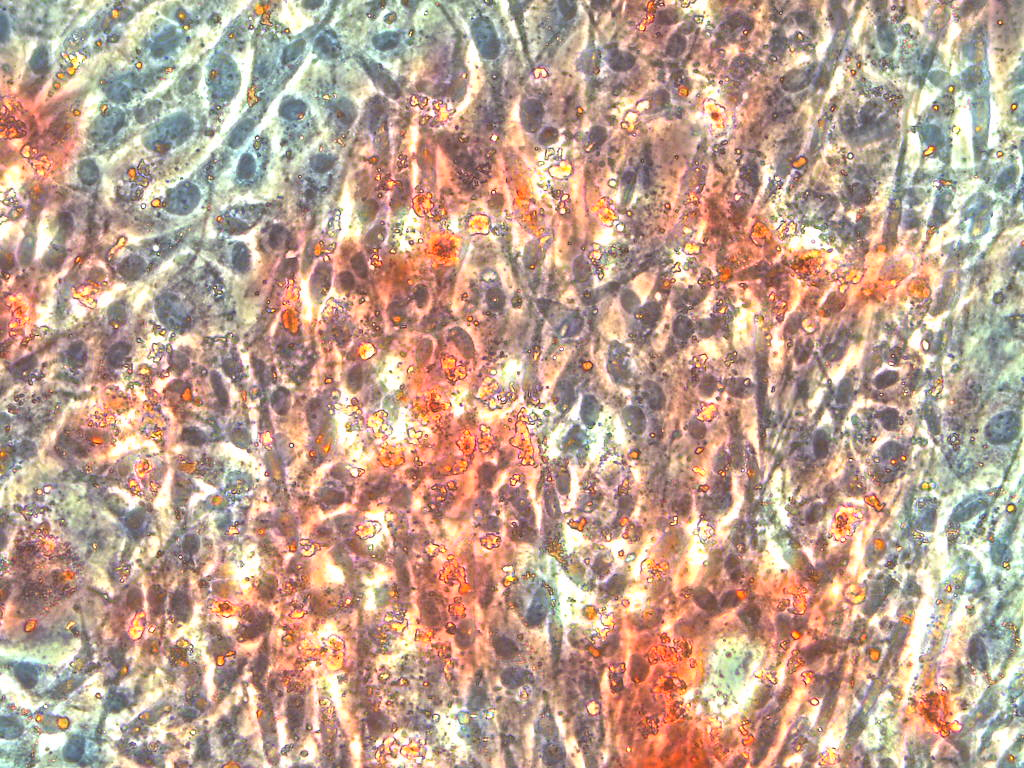

Supplement: Supplemental Material [file KBIE_A_2066047_SM4351.zip › supplementary/Fig5B_Arctiin.jpg]

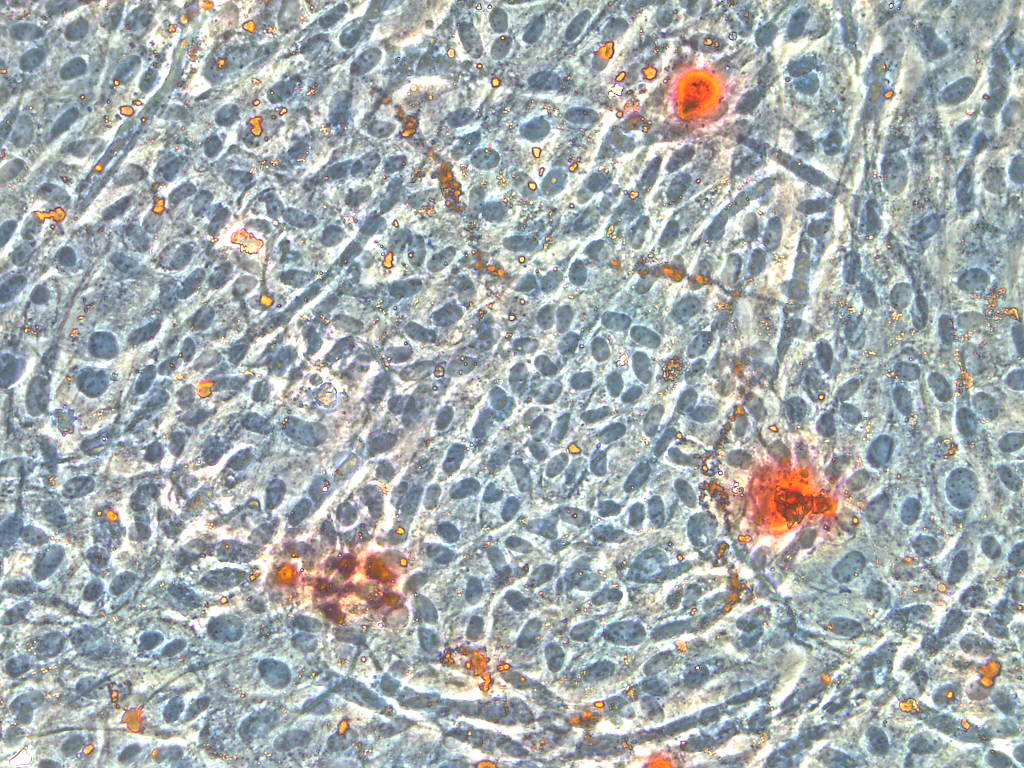

Supplement: Supplemental Material [file KBIE_A_2066047_SM4351.zip › supplementary/Fig5B_Arctiin_shCCND1.jpg]

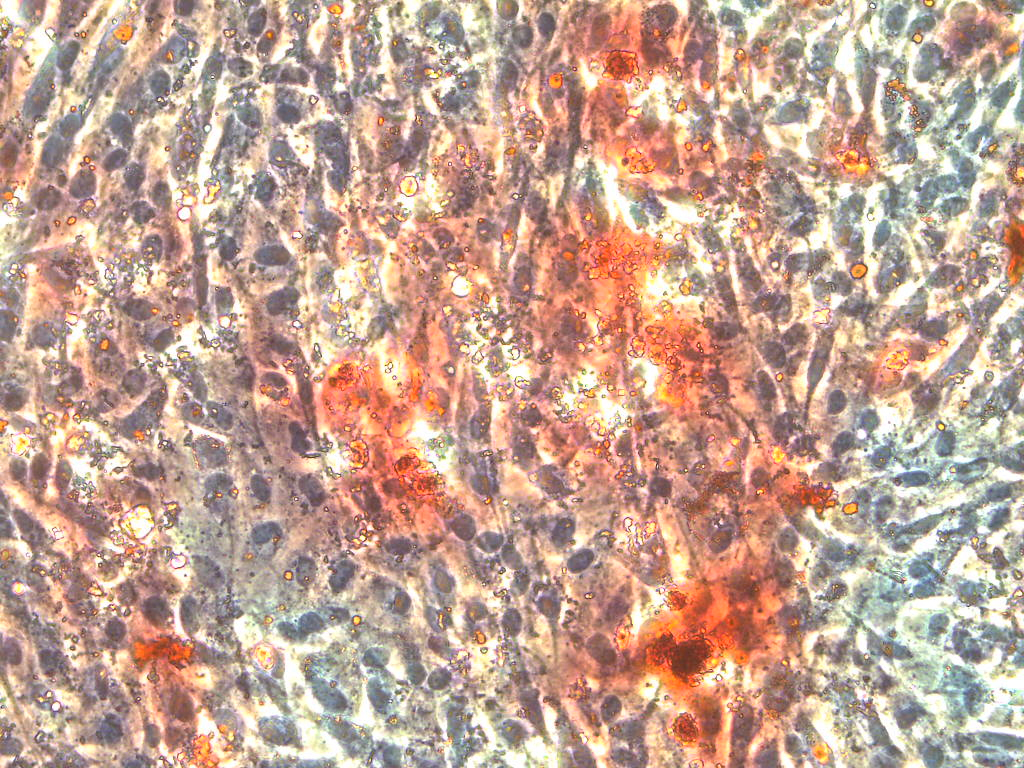

Supplement: Supplemental Material [file KBIE_A_2066047_SM4351.zip › supplementary/Fig5B_Arctiin_shNC.jpg]

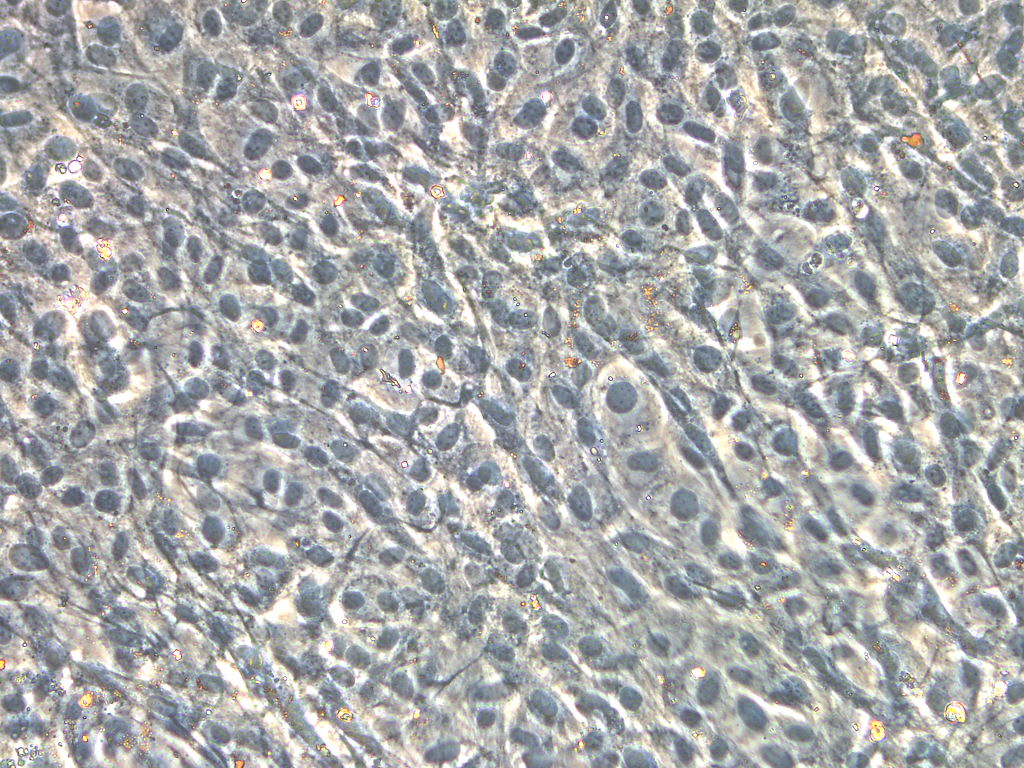

Supplement: Supplemental Material [file KBIE_A_2066047_SM4351.zip › supplementary/Fig5B_Control.jpg]

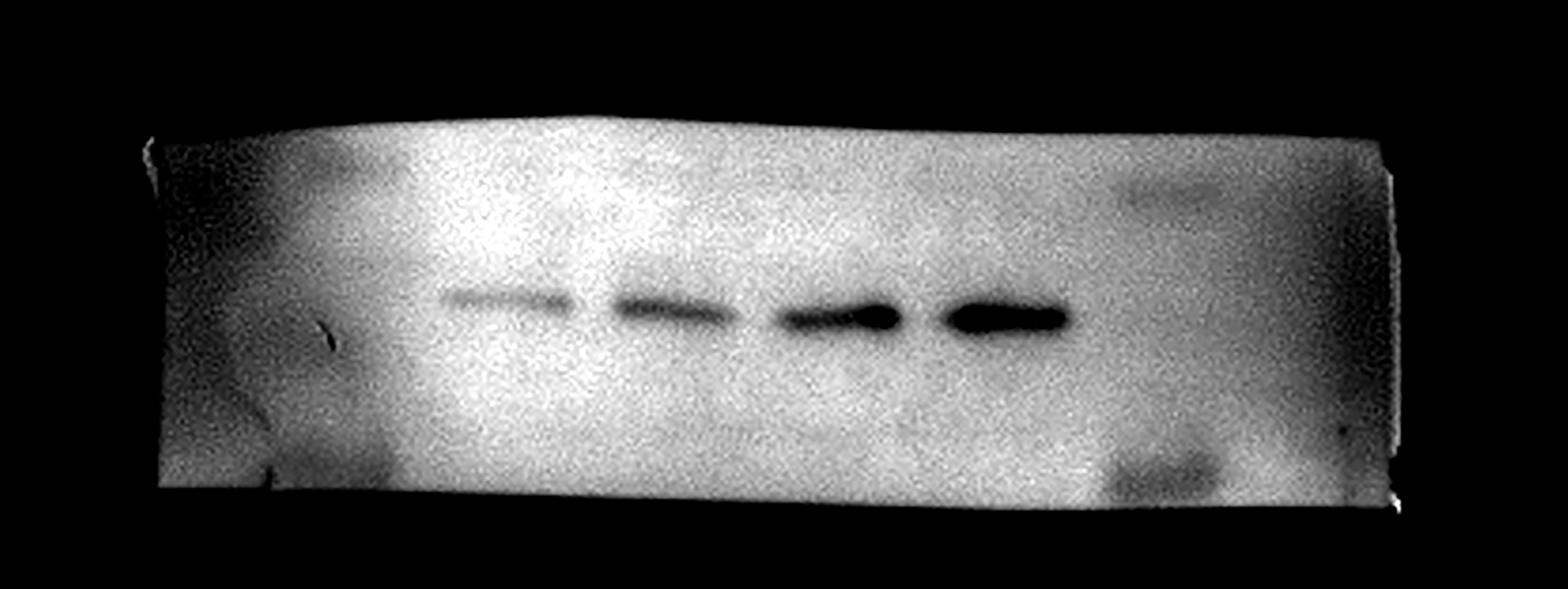

Supplement: Supplemental Material [file KBIE_A_2066047_SM4351.zip › supplementary/Figure1C_COL1A1.tif]

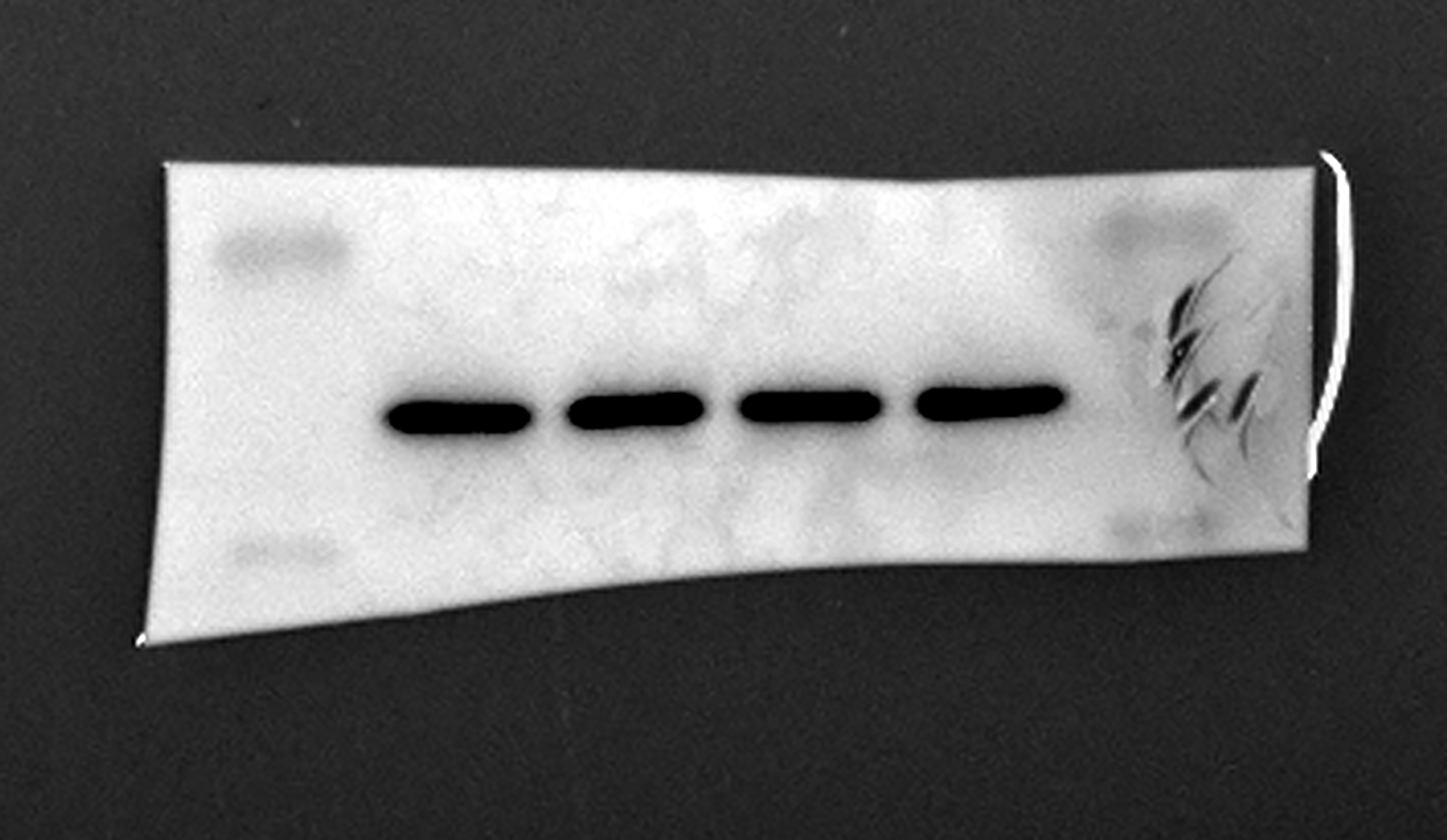

Supplement: Supplemental Material [file KBIE_A_2066047_SM4351.zip › supplementary/Figure1C_GAPDH.tif]

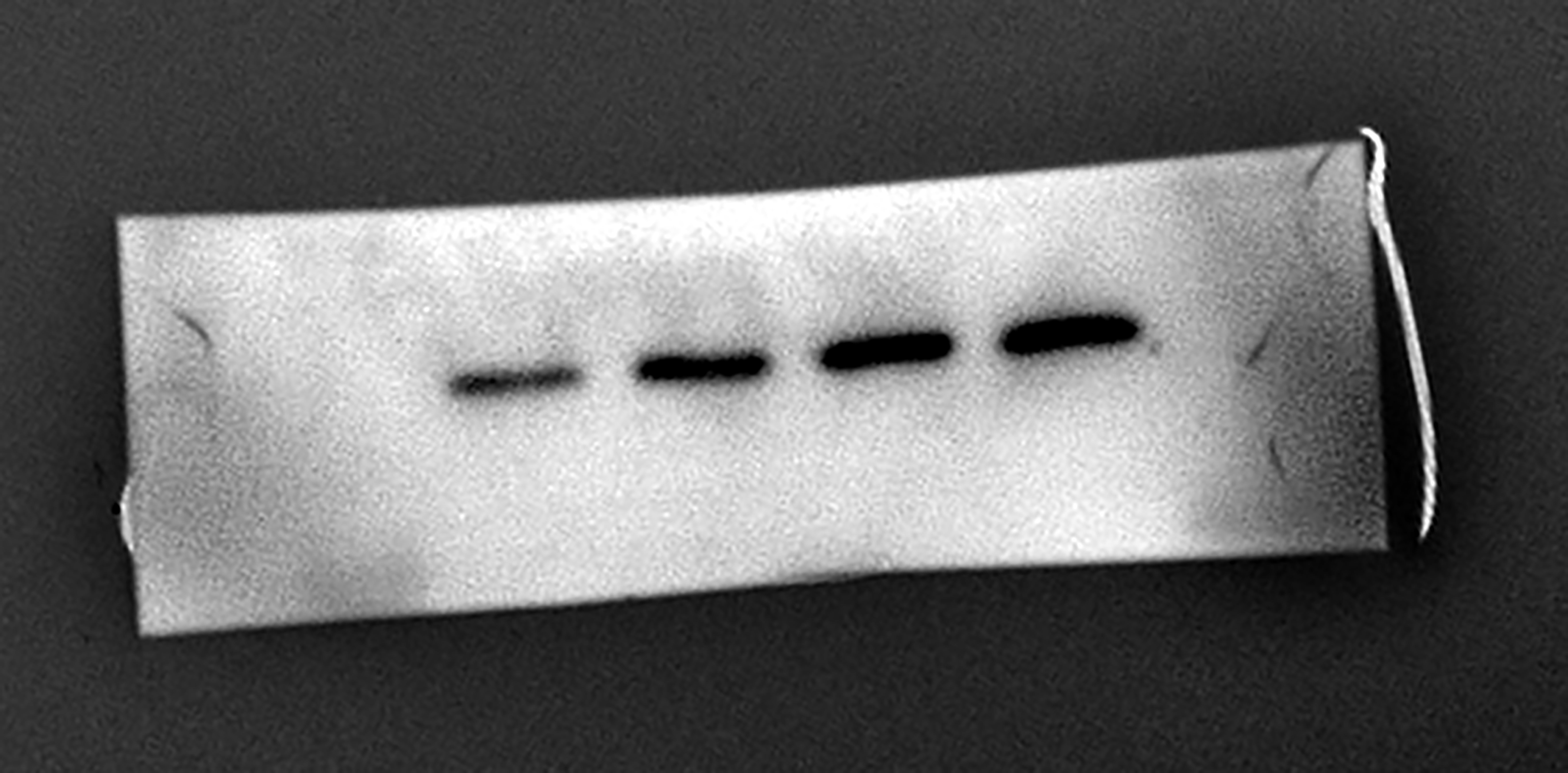

Supplement: Supplemental Material [file KBIE_A_2066047_SM4351.zip › supplementary/Figure1C_OCN.tif]

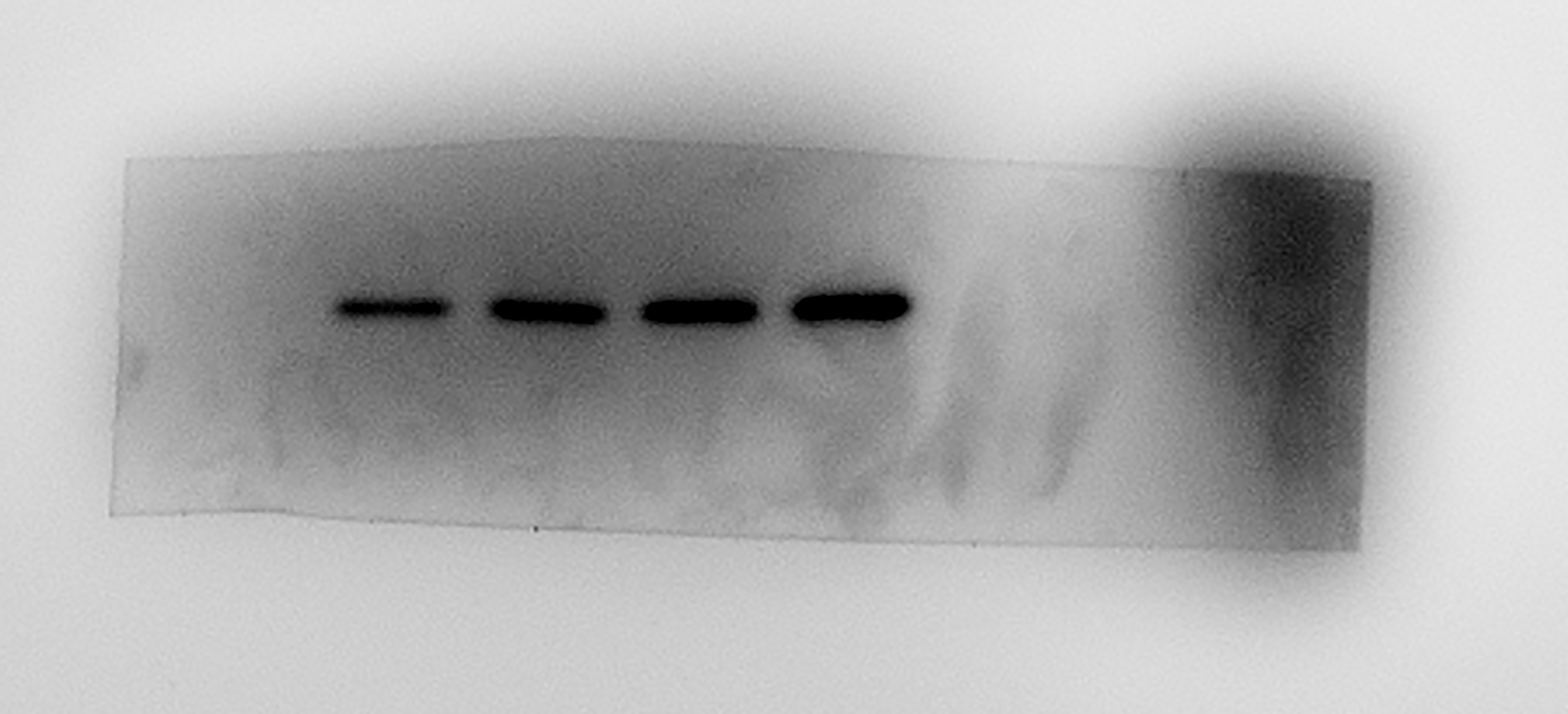

Supplement: Supplemental Material [file KBIE_A_2066047_SM4351.zip › supplementary/Figure1C_OPN.tif]

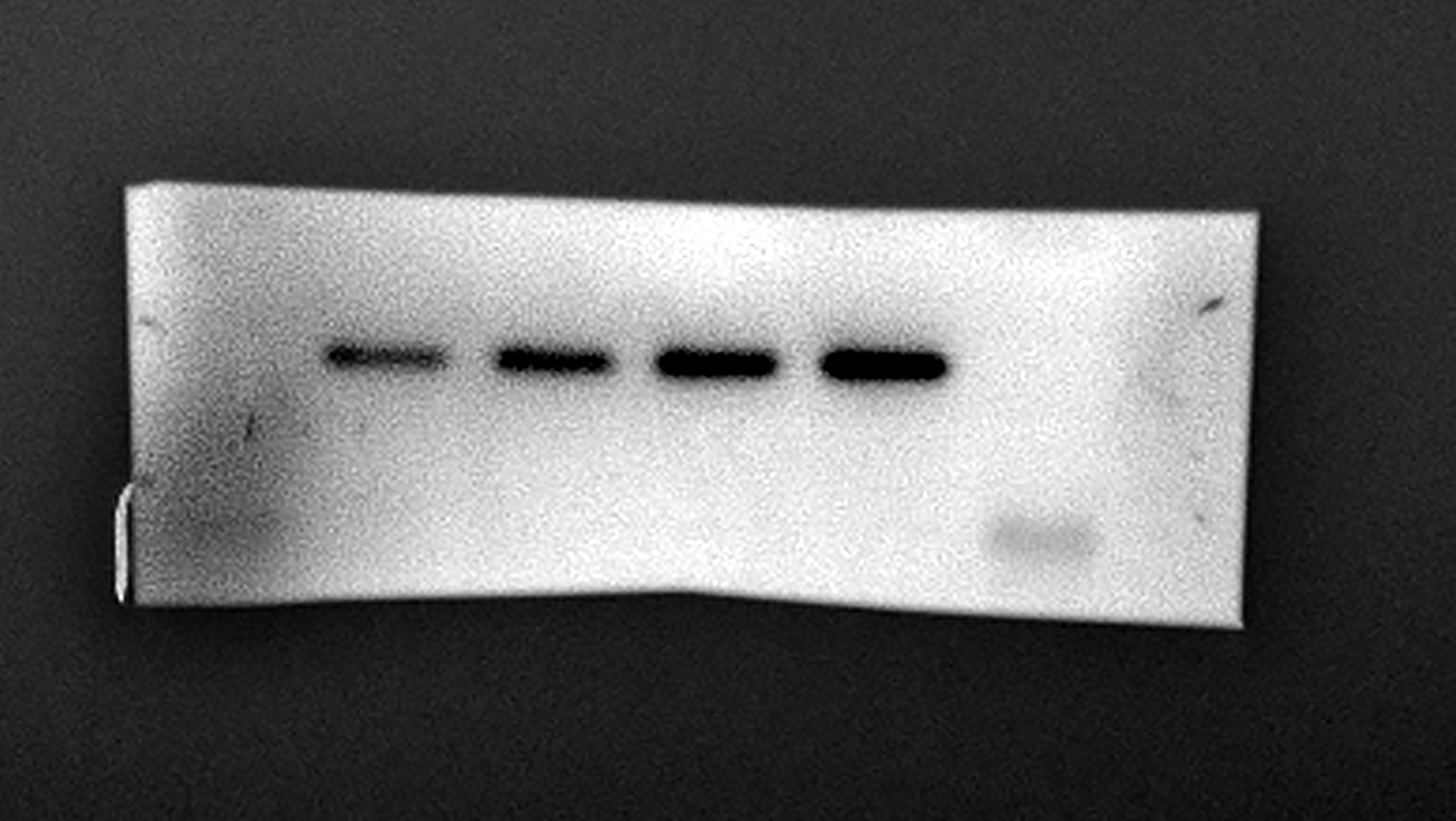

Supplement: Supplemental Material [file KBIE_A_2066047_SM4351.zip › supplementary/Figure1C_RUNX2.tif]

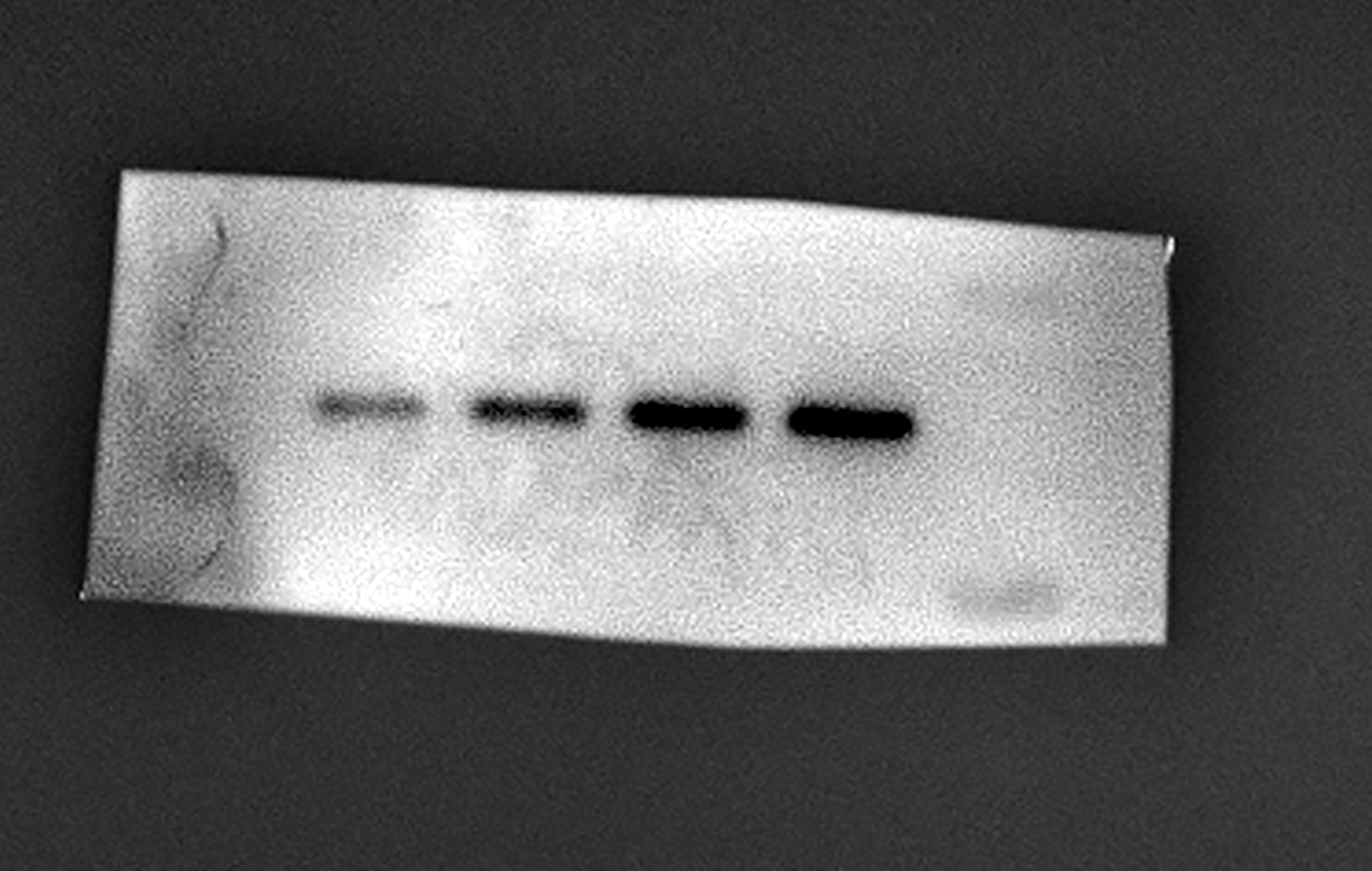

Supplement: Supplemental Material [file KBIE_A_2066047_SM4351.zip › supplementary/Figure3B_Ccnd1.tif]

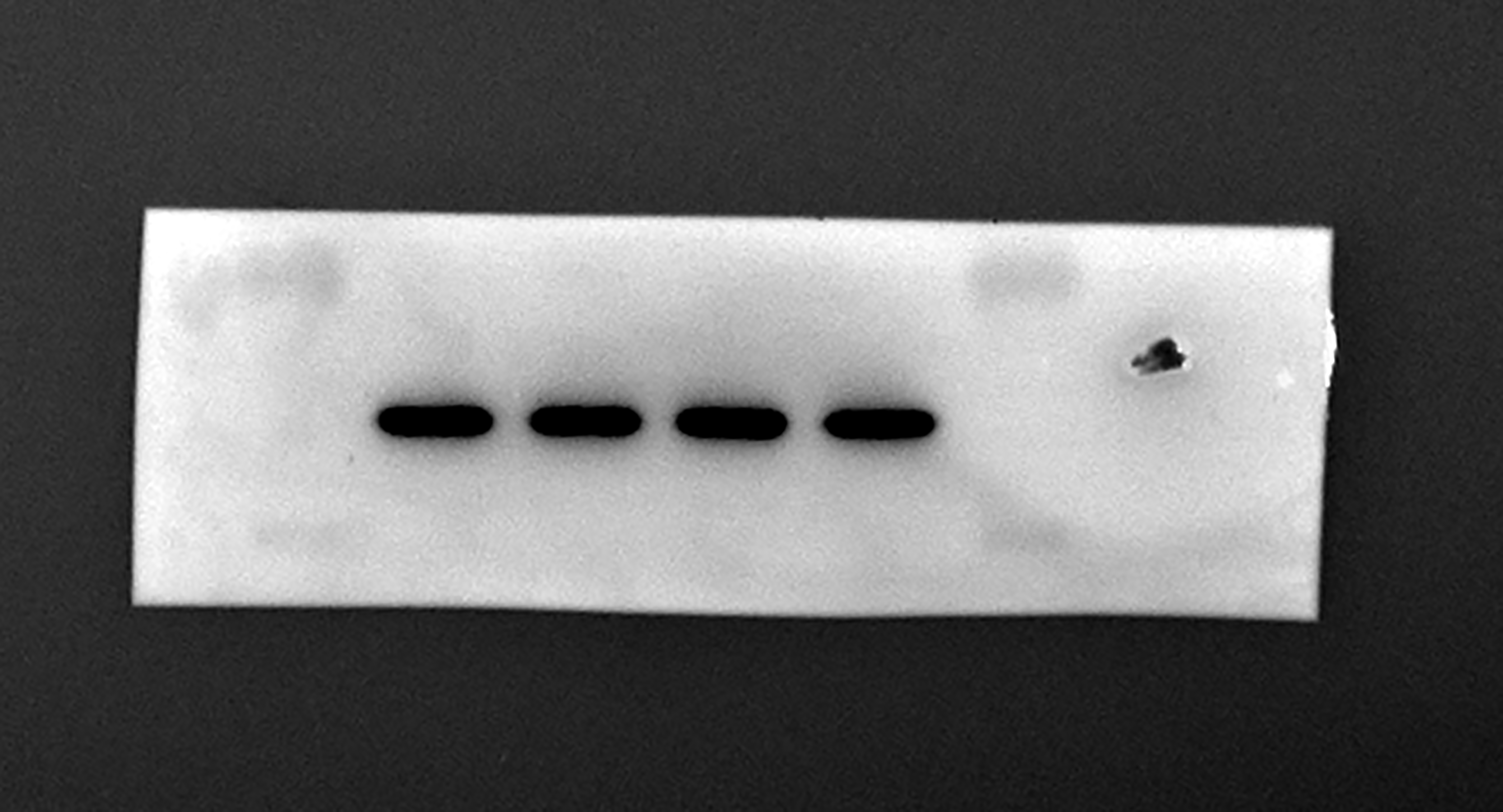

Supplement: Supplemental Material [file KBIE_A_2066047_SM4351.zip › supplementary/Figure3B_GAPDH.tif]

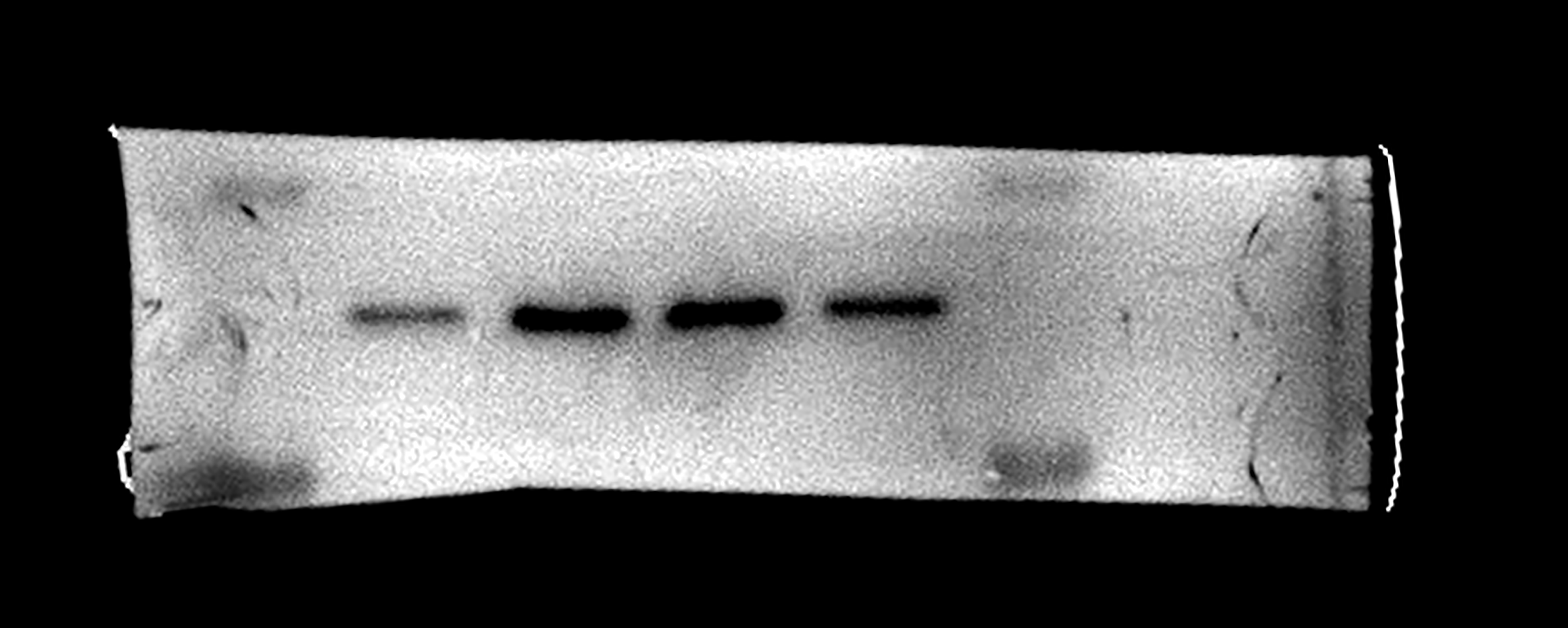

Supplement: Supplemental Material [file KBIE_A_2066047_SM4351.zip › supplementary/Figure4A_Ccnd1.tif]

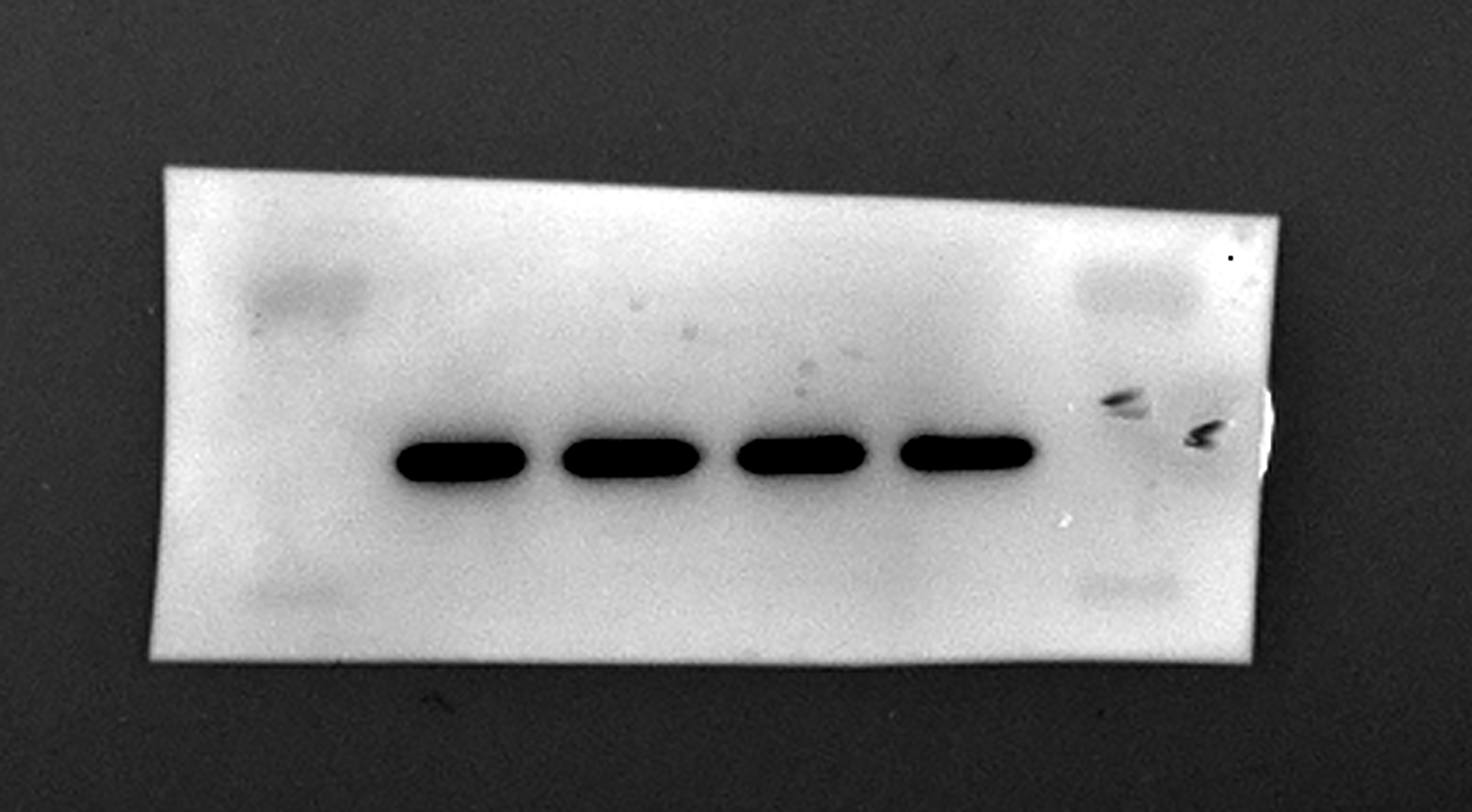

Supplement: Supplemental Material [file KBIE_A_2066047_SM4351.zip › supplementary/Figure4A_GAPDH.tif]

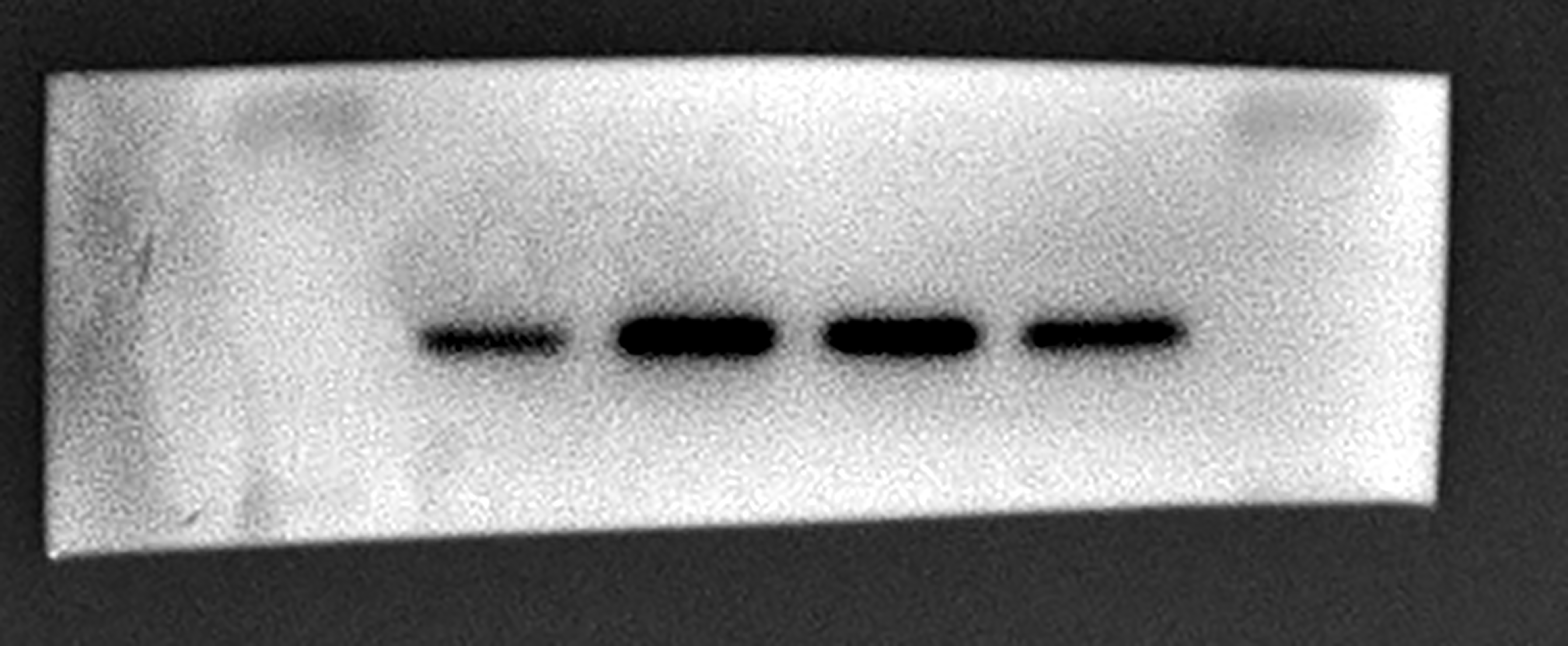

Supplement: Supplemental Material [file KBIE_A_2066047_SM4351.zip › supplementary/Figure4C_COL1A1.tif]

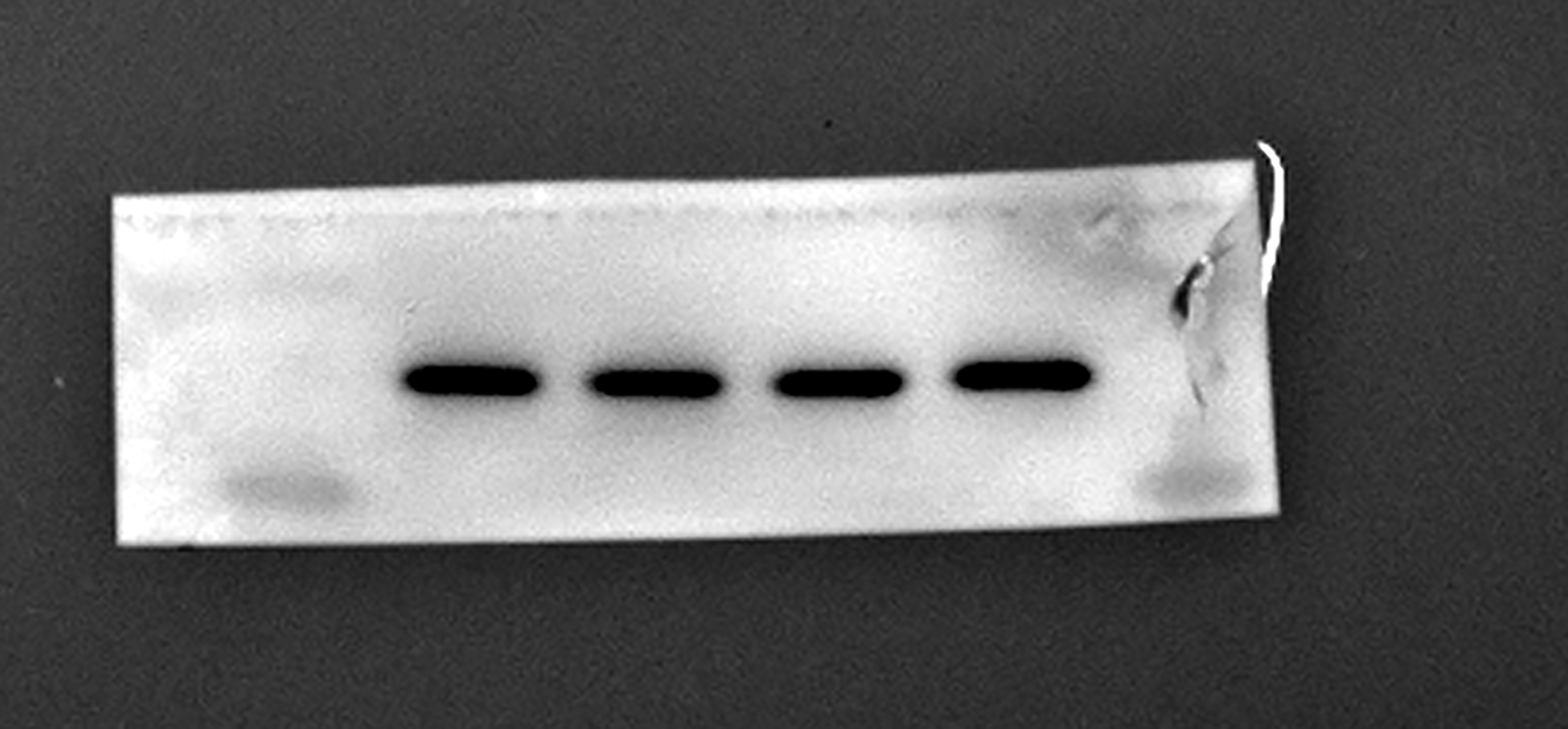

Supplement: Supplemental Material [file KBIE_A_2066047_SM4351.zip › supplementary/Figure4C_GAPDH.tif]

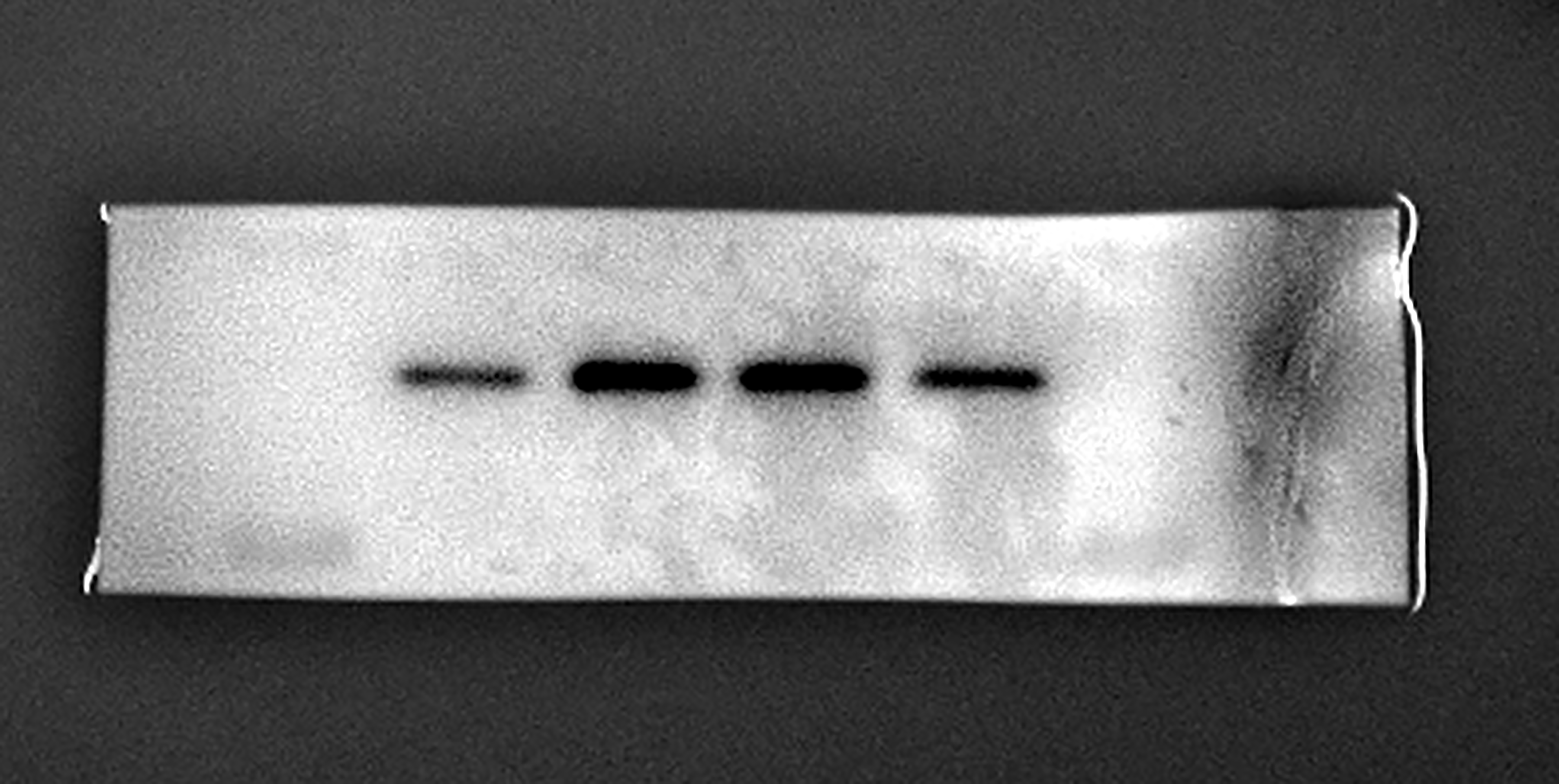

Supplement: Supplemental Material [file KBIE_A_2066047_SM4351.zip › supplementary/Figure4C_OCN.tif]

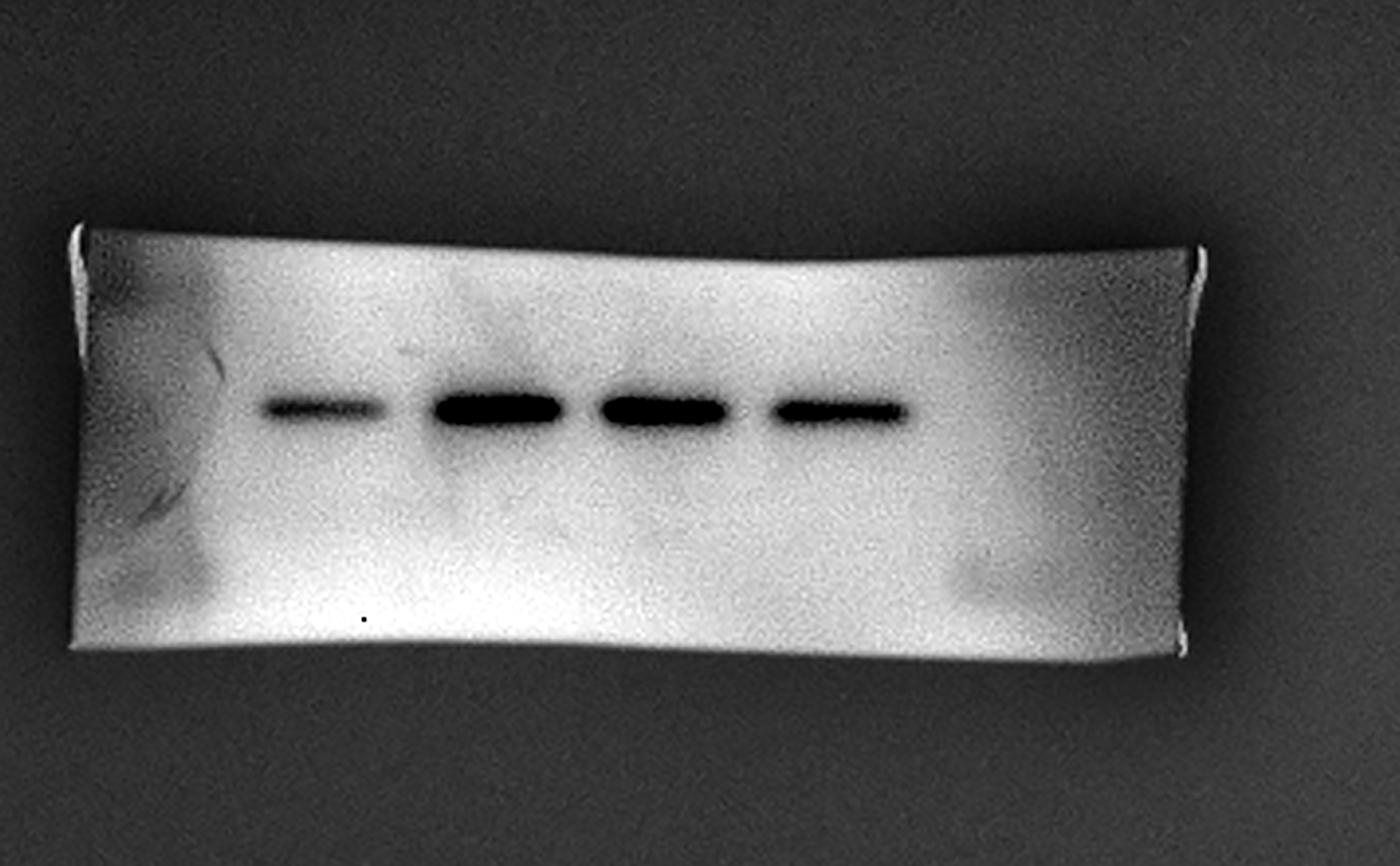

Supplement: Supplemental Material [file KBIE_A_2066047_SM4351.zip › supplementary/Figure4C_OPN.tif]

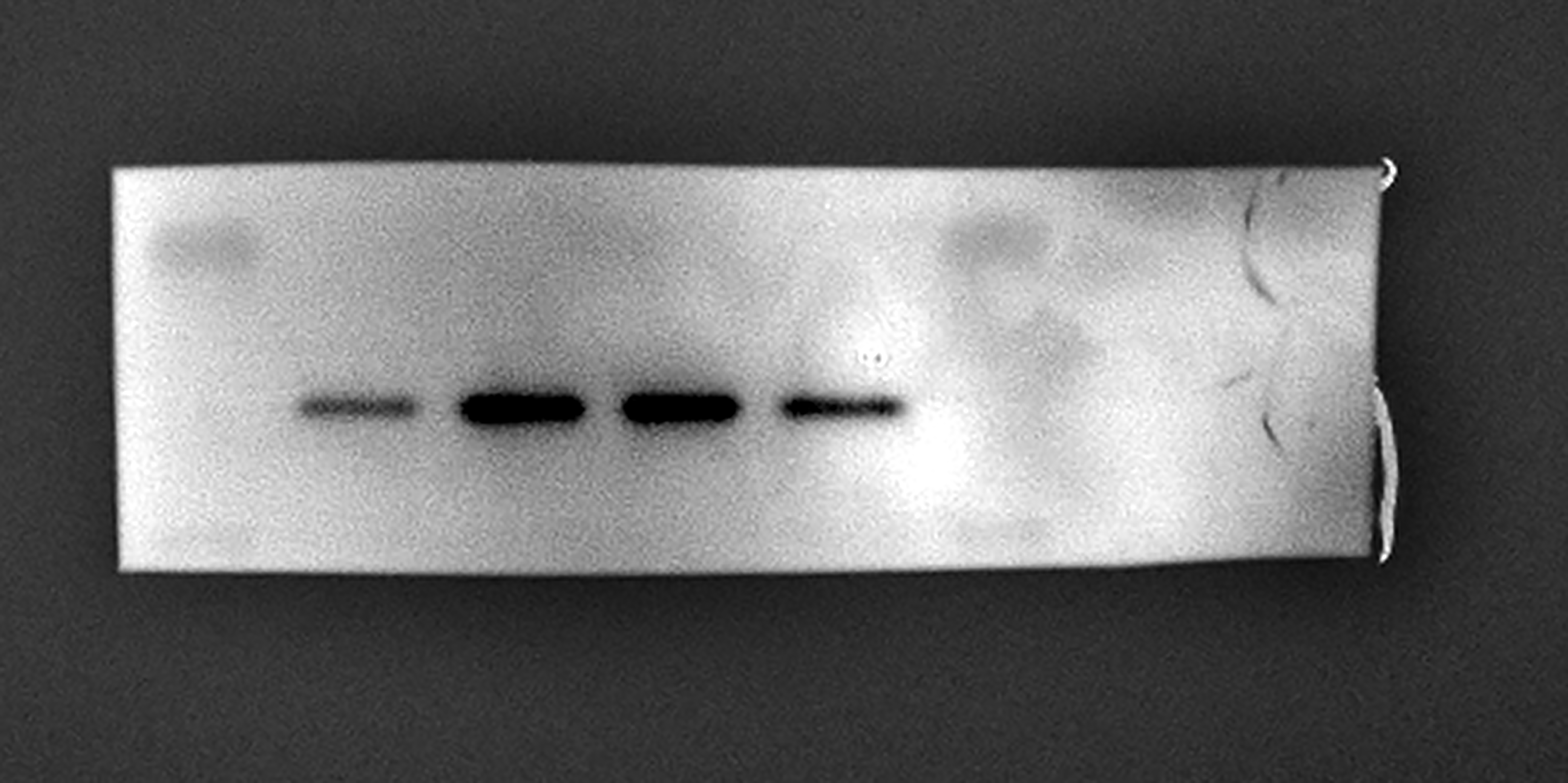

Supplement: Supplemental Material [file KBIE_A_2066047_SM4351.zip › supplementary/Figure4C_RUNX2.tif]
